# Comparison of host endothelial, epithelial and inflammatory response in ICU patients with and without COVID-19: a prospective observational cohort study

**DOI:** 10.1186/s13054-021-03547-z

**Published:** 2021-04-19

**Authors:** Pavan K. Bhatraju, Eric D. Morrell, Leila Zelnick, Neha A. Sathe, Xin-Ya Chai, Sana S. Sakr, Sharon K. Sahi, Anthony Sader, Dawn M. Lum, Ted Liu, Neall Koetje, Ashley Garay, Elizabeth Barnes, Jonathan Lawson, Gail Cromer, Mary K. Bray, Sudhakar Pipavath, Bryan R. Kestenbaum, W. Conrad Liles, Susan L. Fink, T. Eoin West, Laura Evans, Carmen Mikacenic, Mark M. Wurfel

**Affiliations:** 1 Division of Pulmonary, Critical Care and Sleep Medicine, Department of Medicine, University of Washington, 325 9th Avenue, Seattle, WA 98104 USA; 2 Sepsis Center of Research Excellence – University of Washington (SCORE-UW), Seattle, WA USA; 3 Division of Nephrology, Department of Medicine, Kidney Research Institute, University of Washington, Seattle, USA; 4 Department of Radiology, University of Washington, Seattle, WA USA; 5 Department of Medicine, University of Washington, Seattle, WA USA; 6 Department of Laboratory Medicine and Pathology, University of Washington, Seattle, WA USA; 7 Translational Research, Benaroya Research Institute, Seattle, WA USA

**Keywords:** COVID-19, Critical illness, Acute respiratory distress syndrome, Endothelial dysfunction

## Abstract

**Background:**

Analyses of blood biomarkers involved in the host response to severe acute respiratory syndrome coronavirus 2 (SARS-CoV-2) viral infection can reveal distinct biological pathways and inform development and testing of therapeutics for COVID-19. Our objective was to evaluate host endothelial, epithelial and inflammatory biomarkers in COVID-19.

**Methods:**

We prospectively enrolled 171 ICU patients, including 78 (46%) patients positive and 93 (54%) negative for SARS-CoV-2 infection from April to September, 2020. We compared 22 plasma biomarkers in blood collected within 24 h and 3 days after ICU admission.

**Results:**

In critically ill COVID-19 and non-COVID-19 patients, the most common ICU admission diagnoses were respiratory failure or pneumonia, followed by sepsis and other diagnoses. Similar proportions of patients in both groups received invasive mechanical ventilation at the time of study enrollment. COVID-19 and non-COVID-19 patients had similar rates of acute respiratory distress syndrome, severe acute kidney injury, and in-hospital mortality. While concentrations of interleukin 6 and 8 were not different between groups, markers of epithelial cell injury (soluble receptor for advanced glycation end products, sRAGE) and acute phase proteins (serum amyloid A, SAA) were significantly higher in COVID-19 compared to non-COVID-19, adjusting for demographics and APACHE III scores. In contrast, angiopoietin 2:1 (Ang-2:1 ratio) and soluble tumor necrosis factor receptor 1 (sTNFR-1), markers of endothelial dysfunction and inflammation, were significantly lower in COVID-19 (*p* < 0.002). Ang-2:1 ratio and SAA were associated with mortality only in non-COVID-19 patients.

**Conclusions:**

These studies demonstrate that, unlike other well-studied causes of critical illness, endothelial dysfunction may not be characteristic of severe COVID-19 early after ICU admission. Pathways resulting in elaboration of acute phase proteins and inducing epithelial cell injury may be promising targets for therapeutics in COVID-19.

**Supplementary Information:**

The online version contains supplementary material available at 10.1186/s13054-021-03547-z.

## Background

Coronavirus disease 2019 (COVID-19) is caused by the severe acute respiratory syndrome coronavirus-2 (SARS-CoV-2) and can result in organ dysfunction including acute respiratory distress syndrome (ARDS), severe acute kidney injury (AKI), and death [1–3]. While only a minority of COVID-19 patients will become critically ill, these patients take up a disproportionate share of intensive care unit (ICU) resources and experience high mortality [1, 3]. Our previous work has defined markers of endothelial dysfunction as critical determinants of organ failure and mortality among ICU patients, but it is unclear whether COVID-19 shares this pathophysiology [4–6]. A clear understanding of the shared and unique characteristics of COVID-19-related critical illness could help to inform the development of novel therapeutics and the repurposing of existing interventions.

Previous reports have suggested that severe COVID-19 is characterized by derangements in inflammatory, endothelial and epithelial cell injury pathways [7]. However, comparisons to other critically ill populations are under-reported. For example, comparisons of plasma biomarkers of endothelial function have largely focused on COVID-19 alone [8], comparisons between COVID-19 and healthy controls [9], and comparisons between patients with increasing severity of COVID-19 [10]. While these studies have advanced our knowledge, the findings are conflicting and the study designs prevent discriminating differences specific to the host response to severe SARS-CoV-2 infection versus a general signature of critical illness. Thus, it is unknown whether inflammatory, endothelial and epithelial cell injury pathways are an early marker of the host response to SARS-CoV-2, and, in turn, if therapies that target these pathways to modulate the host response should be preferentially tested in COVID-19.

To address this knowledge gap, we developed the COVID-19 Host Response and Clinical Outcomes (CHROME) study enrolling a prospective cohort of patients admitted to an ICU as persons under investigation (PUIs) for COVID-19. Blood was collected within 24 h and again 3 days after ICU admission. We hypothesized that biomarkers of the host response would be different early after ICU admission in COVID-19 compared to a matched cohort of ICU patients without COVID-19.

## Methods

### Clinical data and ascertainment of outcomes

**Fig. 1:**
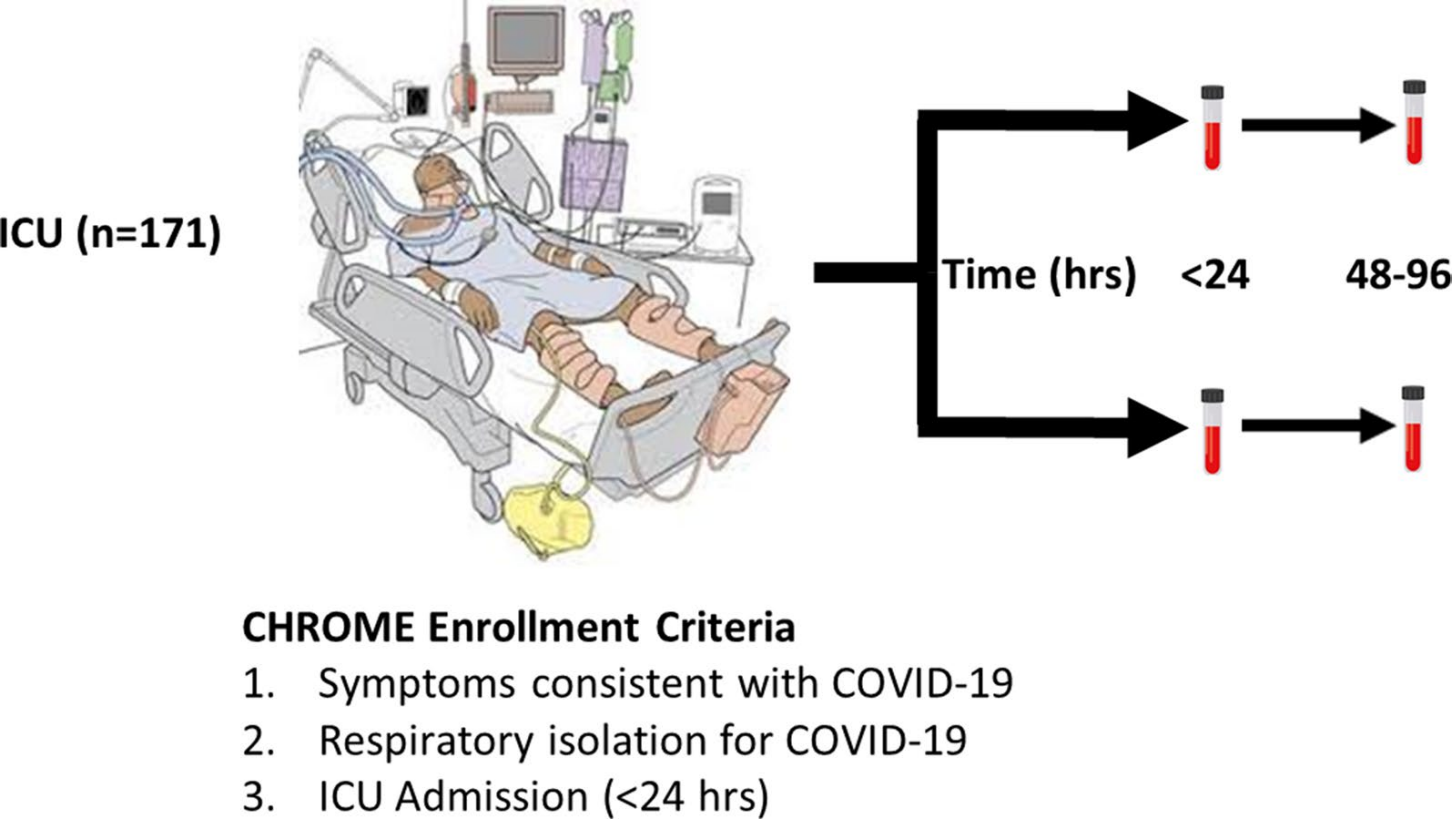
Study overview. Overview of patient cohorts, SARS-CoV-2 positive (COVID-19) and SARS-CoV-2 negative (non-COVID-19) and blood sampling timeline

We prospectively enrolled 171 ICU patients admitted to three University of Washington Hospitals campuses (Montlake, Harborview and Northwest hospitals) with suspicion of COVID-19 infection, including 78 (46%) patients with a confirmed SARS-CoV-2 infection and 93 (56%) with a negative SARS-CoV-2 test in the CHROME cohort (UW IRB: 9763 and 6878) (Fig. 1). Study enrollment started on April 2nd, 2020 and patients included in this analysis were enrolled until September 14th, 2020. All patients included in this analysis had complete data ascertainment. All patients had symptoms consistent with COVID-19, were placed under respiratory isolation by the treating physician (PUI) and had a nasopharyngeal swab sent for SARS-CoV-2. A confirmed case of COVID-19 was defined by a positive result on a reverse-transcriptase–polymerase-chain-reaction (RT-PCR) assay. The primary aim of our study was to determine whether plasma biomarkers of key pathways in sepsis were different between COVID-19 and non-COVID-19 critically ill patients. Our secondary aim was to determine whether differentially expressed plasma biomarkers were associated with clinical outcomes, such as mortality, AKI and ARDS, in COVID-19 and non-COVID patients.

Clinical data was abstracted from the electronic medical record into standardized case report forms. Data to calculate the APACHE III score was collected in the first 24 h of ICU admission [11]. We also determined patient severity on study enrollment using the 8-point ordinal scale as previously reported [12]. Trained research coordinators identified the initiation of inpatient dialysis (hemodialysis or continuous renal replacement therapy), thrombosis, shock, ARDS and death by chart review and all outcomes were adjudicated until death or hospital discharge. ARDS was categorized by Berlin Criteria [13], reflecting each individual’s worst oxygenation level (based on the ratio of the inspired FIO_2_ and the arterial PaO_2_) in combination with the adjudication of chest radiographs by an Attending Radiologist (SP). AKI was defined as an increase ≥ 0.3 mg/dL in 48 h and/or ≥ 50% in serum creatinine concentrations measured during hospitalization compared to a ‘baseline’ serum creatinine value measured at CHROME study enrollment or need for new dialysis. Severe AKI was defined as doubling of serum creatinine compared to a ‘baseline’ serum creatinine value or need for new dialysis after study enrollment [14].

### Sample processing and protein analyses

Peripheral blood was collected into EDTA anti-coagulant tubes within 24 h of ICU admission and, for a subset, again at day 3 who were still in the ICU. Plasma was isolated by centrifugation (10 min, 3000 rpm, room temperature). Biomarkers were chosen a priori given literature supporting their use for querying the selected pathways and availability of established immunoassays [5, 15, 16]. We measured 22 biomarkers, which included markers of *endothelial dysfunction/activation* [angiopoietin-1 (Ang-1), angiopoietin-2 (Ang-2), basic fibroblast growth factor (bFGF), placental growth factor (PIGF), soluble fms-like tyrosine kinase 1 (sFlt-1), soluble Tie-2 (sTie2), vascular endothelial growth factors (VEGF) A, C, and D, Eotaxin-1, Eotaxin-3, intercellular adhesion molecule 1 (sICAM-1), vascular cell adhesion molecule (sVCAM)], *inflammation* [interleukin-6 (IL-6), interleukin-8 (IL-8), soluble tumor necrosis factor receptor-1 (sTNFR-1), tumor necrosis factor-α (TNF-α), c-reactive protein (CRP), serum amyloid A (SAA)] and *epithelial injury/apoptosis/innate immune activation* [soluble receptor for advanced glycation end products (sRAGE), soluble Fas (sFAS) and soluble triggering receptor expressed by myeloid cells 1 (sTREM-1)]. Additional methods of biomarker measurements and the coefficient of variations can be found in the supplement and Additional file 1: Table S1.

### Statistical analyses

For baseline characteristics, we report continuous variables as mean ± SD or median and interquartile range (IQR) and categorical variables as number and percent. Since biomarker concentrations can be skewed all biomarkers were log_2_ transformed. Height data was missing for five participants and we used multiple imputation using chained equations, combining across imputations using Rubin’s rules. To determine the risk of clinical outcomes, we performed relative risk (RR) regression using a Poisson model and robust standard error estimates to test for associations between clinical outcomes, such as ARDS, severe AKI (Stage 2 or 3), dialysis and death and COVID-19 status. We selected adjustment variables a priori on the basis of biologic plausibility and prior literature suggesting these variables may confound associations between plasma biomarkers and clinical outcomes [17, 18]. All comparisons were adjusted for age, sex, body mass index (BMI), APACHE III scores and Charlson comorbidity index.

To compare the fold changes in biomarker concentrations between COVID-19 and non-COVID-19 patients, we used linear regression with robust Huber-White standard errors of the log-transformed plasma biomarker on COVID-19 status. Analyses were adjusted for age, sex, BMI and APACHE III scores. To control type 1 error from multiple hypothesis testing, we used a Bonferroni corrected *p* value of 2.2 × 10^–3^ (0.05/22 biomarkers). We also performed subgroup analyses to explore whether the magnitude of associations between plasma biomarkers and COVID-19 populations differed by primary ICU admission diagnosis. We only carried forward biomarkers that were significantly different using a Bonferroni-corrected threshold between COVID-19 and non-COVID-19. We next used a fold ratio of biomarker concentrations from day 1 to day 3 in the subset of patients who had blood collected at both timepoints. We tested the change in fold ratio over time stratified by COVID-19 status.

In secondary analyses, we tested whether plasma biomarkers were differentially associated with clinical outcomes based on COVID-19 status. We used RR regression to identify associations between biomarker concentrations and clinical outcomes stratified by COVID-19 status. We present univariate and multivariate associations between plasma biomarkers and clinical outcomes as RR per doubling of the plasma biomarker. All analyses were performed in R, version 3.6.2 (R Project for Statistical Computing).

## Results

### Participant characteristics between COVID-19 and non-COVID-19 ICU patients

Among 171 patients admitted to an ICU as a PUI, 78 patients ultimately tested positive for SARS-CoV-2 (COVID-19) and 93 tested negative (non-COVID-19). Baseline characteristics are provided in Table 1. Critically ill COVID-19 and non-COVID-19 patients had a similar mean (± SD) age (54.3 ± 17.5 and 54.8 ± 16.9 years, respectively) and BMI (29.6 ± 7.3 and 30.4 (± 11.2), respectively). Patients with COVID-19 were more likely to be men (72% vs. 59%) and be of Hispanic ethnicity (45% vs 8%).

**Table 1:** Baseline clinical characteristics, COVID-19 therapies, and outcomes

Characteristics	ICU COVID-19 Negative (*N* = 93)	ICU COVID-19 Positive (*N* = 78)	*p *value
Mean age—year	54.8 (± 16.9)	54.3 (± 17.5)	0.85
Male—no. (%)	55 (59)	56 (72)	0.12
Race—no. (%)			
American Indian/Alaska Native	3 (3)	2 (3)	0.25
Asian	5 (5)	7 (9)	
Black/African American	17 (18)	6 (8)	
White	59 (63)	56 (72)	
Unknown/other	9 (10)	4 (5)	
Ethnicity—no. (%)			
Hispanic or Latino	7 (8)	35 (45)	< 0.001
Not Hispanic or Latino	79 (85)	42 (54)	
Unknown	7 (8)	1 (1)	
Mean body mass index—kg/m^2^	30.4 (± 11.2)	29.6 (± 7.3)	0.26
Coexisting disorder—no. (%)			
Asthma	21 (23)	10 (13)	0.15
Cerebrovascular Disease	8 (9)	8 (10)	0.92
Chronic Kidney Disease	26 (28)	11 (14)	0.04
Chronic Obstructive Pulmonary Disease	23 (25)	3 (4)	< 0.001
Coronary Artery Disease	15 (16)	8 (10)	0.37
Congestive Heart Failure	18 (19)	8 (10)	0.15
Diabetes mellitus	29 (31)	22 (28)	0.73
Hypertension	51 (55)	35 (45)	0.25
Study Enrollment ACTT-1 8-Point Ordinal Scale, *n* (%)			
4 (Hospitalized, no O_2_ therapy, requiring ongoing medical care)	16 (17)	16 (21)	0.25
5 (Hospitalized, any supplemental 0_2_)	18 (19)	14 (18)	
6 (Non-invasive ventilation or high flow nasal cannula)	15 (16)	13 (17)	
7 (invasive mechanical ventilation or extracorporeal membrane oxygenation)	44 (47)	35 (45)	
Primary ICU Admission Diagnosis, no. (%)			
Pneumonia or respiratory distress/failure	70 (75)	58 (74)	0.39
Sepsis^a^	9 (10)	4 (5)	
Other	14 (15)	16 (21)	
ARDS	23 (25)	28 (36)	0.12
APACHE III Score	80.8 (± 29.5)	70.5 (± 28.7)	0.02
Admission PaO_2_:FIO_2_ ratio—median (IQR)^b^	50 (31–70)	70 (45–80)	0.17
Charlson Comorbidity Index	4.7 (2.9)	3.4 (2.3)	0.001
COVID-19 specific therapies, no. (%)			
Convalescent plasma	0 (0)	16 (21)	< 0.001
Remdesivir	0 (0)	12 (15)	< 0.001
Hydroxychloroquine	0 (0)	11 (14)	< 0.001
Tocilizumab	0 (0)	6 (8)	0.01
Dexamethasone of 6 mg or equivalent glucocorticoid dose	31 (33)	31 (40)	0.39
Length of hospital stay, median (IQR), days	15.3 (± 16.4)	23.0 (± 23.6)	0.02

Entries are mean (± SD) for continuous variables, or *N* (%) for categorical variables

*APACHE III* acute physiology and chronic health evaluation, *ACTT-1* Adaptive COVID-19 Treatment Trial

^a^Diagnosis of sepsis included not having a primary diagnosis of respiratory failure or pneumonia and having one of the following ICU admission diagnoses: sepsis, septic Shock, necrotizing soft tissue infection, bacteremia, cellulitis, urinary tract infection and abscess

^b^Data on admission PaO_2_:FIO_2_ ratio were missing for 17 patients without COVID-19 and 14 patients with COVID-19 who received mechanical ventilation

The primary ICU admission diagnosis of respiratory failure or pneumonia was approximately 75% in both groups. Causes of respiratory failure in the non-COVID-19 population included bacterial pneumonia, aspiration pneumonia, chronic obstructive pulmonary disease exacerbation and others (Additional file 1: Table S2). Viral causes of pneumonia were uncommon in the non-COVID-19 group. Scores for the 8-point ordinal scale were similar in patients with and without COVID-19 (Table 1). A similar proportion of patients in both COVID-19 and non-COVID-19 groups were receiving oxygen therapy via non-invasive positive pressure ventilation or high flow nasal cannula (17% and score 6) and 46% were receiving invasive mechanical ventilation or extracorporeal membrane oxygenation (score 7) at study enrollment. Mean APACHE III scores were higher in non-COVID-19 (80.8 ± 29.5) than COVID-19 patients (70.5 ± 28.7).

### Rates of ARDS, AKI, thromboembolism and death were similar between COVID-19 and non-COVID-19 patients

Rates of ARDS (COVID-19: 36% vs. non-COVID-19: 25%), severe AKI (COVID-19: 15% vs. non-COVID-19: 12%), new dialysis (COVID-19: 10% vs non-COVID-19: 6%), thromboembolism (COVID-19: 14% vs. non-COVID-19: 15%), and hospital mortality (COVID-19: 26% vs. non-COVID-19: 22%) were similar between groups (Table 2). In addition, the risk of ARDS, severe AKI, new dialysis, thromboembolism and hospital mortality were not significantly different in unadjusted and adjusted analyses. Length of hospital stay was significantly longer in COVID-19 patients. Patients with COVID-19 had an adjusted length of stay of 8.6 days longer than non-COVID-19 patients (95% CI 1.95–15.2, *p* = 0.02).

**Table 2:** Risk of clinical outcomes in ICU patients with and without COVID-19

Clinical outcomes	ICU COVID-19 Negative (*N* = 93)	ICU COVID-19 Positive (*N* = 78)	Unadjusted relative risk (95% CI)	*p* value	Model 1 relative risk (95% CI)	*p* value	Model 2 relative risk (95% CI)	*p* value
In-hospital mortality	20 (22)	20 (26)	1.19 (0.69, 2.05)	0.53	1.24 (0.74, 2.10)	0.41	1.50 (0.93, 2.41)	0.10
ARDS	23 (25)	28 (36)	1.45 (0.91, 2.30)	0.11	1.39 (0.87, 2.22)	0.17	1.32 (0.87, 1.98)	0.19
Shock requiring vasopressor therapy	43 (46)	39 (50)	1.08 (0.79, 1.48)	0.62	1.07 (0.79, 1.45)	0.67	1.14 (0.84, 1.54)	0.40
AKI^a^	24 (26)	23 (29)	1.06 (0.66, 1.71)	0.81	0.96 (0.60, 1.54)	0.86	1.04 (0.64, 1.69)	0.87
Severe AKI^a^	11 (12)	12 (15)	1.21 (0.57, 2.57)	0.63	1.08 (0.50, 2.31)	0.85	1.13 (0.51, 2.52)	0.77
New dialysis^a^	6 (6)	8 (10)	1.48 (0.54, 4.06)	0.45	1.31 (0.48, 3.58)	0.60	1.60 (0.63, 4.09)	0.33
Thromboembolism	14 (15)	11 (14)	0.94 (0.45, 1.94)	0.86	0.83 (0.40, 1.71)	0.62	0.60 (0.28, 1.27)	0.18

*ARDS* acute respiratory distress syndrome, *APACHE III* acute physiology and chronic health evaluation, *AKI* acute kidney injury. Model 1 adjusted: age, gender, body mass index and APACHE III. Model 2 includes model 1 variables and Charlson comorbidity index. Relative risk regression with 95% confidence intervals. Relative risk (RR) estimates are comparing COVID-19 to non-COVID-19 patients

^a^All AKI outcomes excluded patients on hemodialysis prior to study enrollment, which included 3 patients in the COVID-19 group and 10 patients in the non-COVID-19 group

### Correlations in key plasma biomarkers reflecting epithelial cell injury, inflammation and endothelial activation/dysfunction between ICU patients with and without COVID-19

**Fig. 2:**
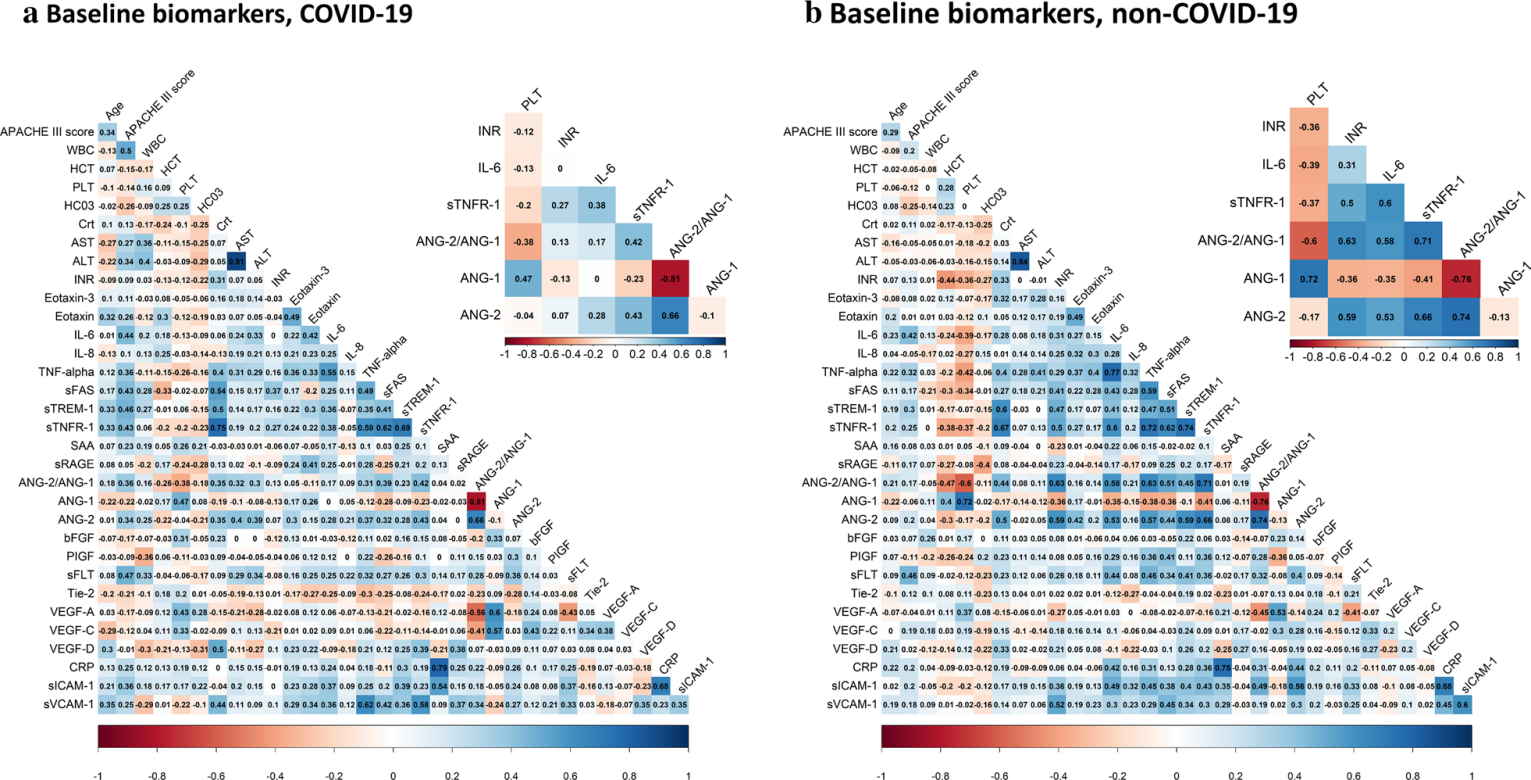
Plasm biomarker correlations. **a** Correlations between plasma biomarkers and key clinical variables (age and APACHE III scores) in COVID-19. Insert highlights key inflammatory, endothelial and coagulation biomarkers. **b** Correlations between plasma biomarkers and key clinical variables (age and APACHE III scores) in non-COVID-19. Insert highlights key inflammatory, endothelial and coagulation biomarkers. Colors represent the correlation with scale indicating value of Pearson’s *r* correlation

Among non-COVID-19 patients, clinical biomarkers of coagulation (international normalized ratio (INR)) were moderately correlated with endothelial markers (Ang-2 and Ang-2:1 ratio (range of correlation 0.59–0.64). In contrast, in COVID-19 patients, INR was minimally correlated with endothelial markers (range of correlation 0.07–0.16). In non-COVID-19 patients, platelet counts and Ang-1 concentrations were highly correlated (*r* = 0.72) while the correlation was much weaker in COVID-19 (*r* = 0.47). In non-COVID-19 patients, inflammatory biomarkers, such as IL-6 and sTNFR-1, were moderate to highly correlated with Ang-2 and Ang-2:1 ratio (range of correlation 0.53–0.71). In contrast, COVID-19 patients these inflammatory biomarkers were minimally correlated with endothelial biomarkers (range of correlation 0.17–0.42). (Fig. 2a, b). These findings suggest the relationship between coagulation and endothelial markers of injury may be distinct in subjects based on COVID-19 status. We also compared 13 clinical biomarkers (serum creatinine, white blood cell count and others) between COVID-19 and non-COVID-19. Among the clinical biomarkers, we found that white blood cell count was significantly lower in COVID-19, while platelet counts were significantly higher in COVID-19 compared to non-COVID-19 (Additional file 1: Table S3).

**Fig. 3:**
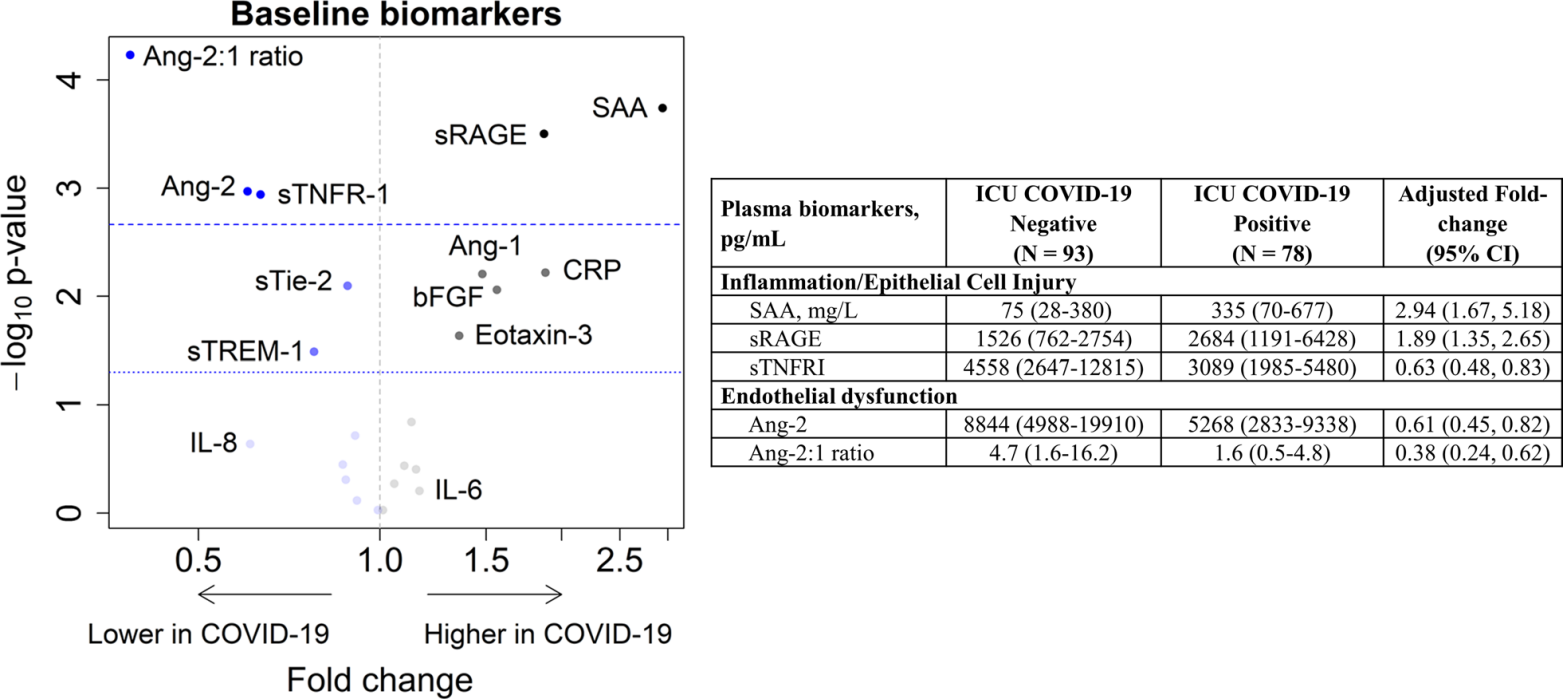
Plasma biomarkers reveal distinct host response between critically ill patients with COVID-19 compared to critically ill controls. Volcano plot of 22 plasma biomarkers analyzed in blood collected within 24 h of ICU admission analyzed in COVID-19 (*n* = 78) relative to non-COVID-19 (*n* = 93) samples. Dashed line indicates Bonferroni-corrected threshold; dotted line indicates nominal 5% significance threshold. Blue dots represent biomarker concentrations that are lower in COVID-19 and gray represent concentrations higher in COVID-19. Estimates are adjusted for age, sex, BMI, and APACHE III score. Table provides plasma biomarkers, median (interquartile range), that significantly differed between ICU populations include angiopoietin-2:1 ratio (Ang-2:1 ratio), soluble receptor for advanced glycation end-products (sRAGE), soluble tumor necrosis factor receptor 1 (sTNFR-1), serum amyloid A (SAA) and angiopoietin-2 (Ang-2)

In order to understand how COVID-19 modulates host response, we compared baseline plasma biomarkers between ICU patients with and without COVID-19 adjusting for age, gender, BMI and APACHE III scores. Among 22 plasma biomarkers, five were significantly different between COVID-19 and non-COVID-19 after Bonferroni correction (Ang-2:1 ratio, Ang-2, sTNFR-1, sRAGE, and SAA) (Fig. 3 and Additional file 1: Table S4). Since corticosteroids could modulate the host response to infection, we completed a sensitivity analysis adjusting for receipt of corticosteroids. We found that while the associations were mildly attenuated, the same five plasma biomarkers (Ang-2:1 ratio, Ang-2, sTNFR-1, sRAGE, and SAA) remained significantly different between COVID-19 and non-COVID-19 patients (Additional file 1: Figure S1).

### Differences in epithelial cell injury and inflammatory pathways between COVID and non-COVID-19

Biomarkers of epithelial cell injury (sRAGE) and acute inflammation (SAA) were higher in COVID-19. Concentrations of sRAGE were approximately twofold greater in COVID-19 (95% CI 1.35–2.65; *p* < 0.001), and concentrations of SAA were 2.9-fold greater in COVID-19 (95% CI 1.67–5.28; *p* < 0.001) than non-COVID-19 patients. In contrast, soluble receptor involved in inflammation (sTNFR-1; adjusted fold change 0.63; 95% CI 0.48–0.83; *p* = 0.001) was significantly lower in COVID-19 than non-COVID-19 patients.

### Differences in endothelial dysfunction/activation pathways between COVID-19 and non-COVID-19

Markers of endothelial dysfunction/activation (Ang-2; adjusted fold change: 0.61; 95% CI 0.45–0.82; *p* = 0.001 and Ang-2:1 ratio; adjusted fold change 0.38; 95% CI 0.24–0.62; *p* < 0.001) were significantly lower in COVID-19 than non-COVID-19 patients. A number of additional biomarkers were nominally significantly different between groups (*p* < 0.05) with Ang-1, bFGF, Eotaxin-3 and CRP being higher in COVID-19, while sTie-2 and sTREM-1 were lower in COVID-19. We carried forward biomarkers passing a Bonferroni-corrected *p *value threshold (Ang-2:1 ratio, Ang-2, SAA, sTNFR-1 and sRAGE) to sensitivity and secondary analyses.

**Fig. 4:**
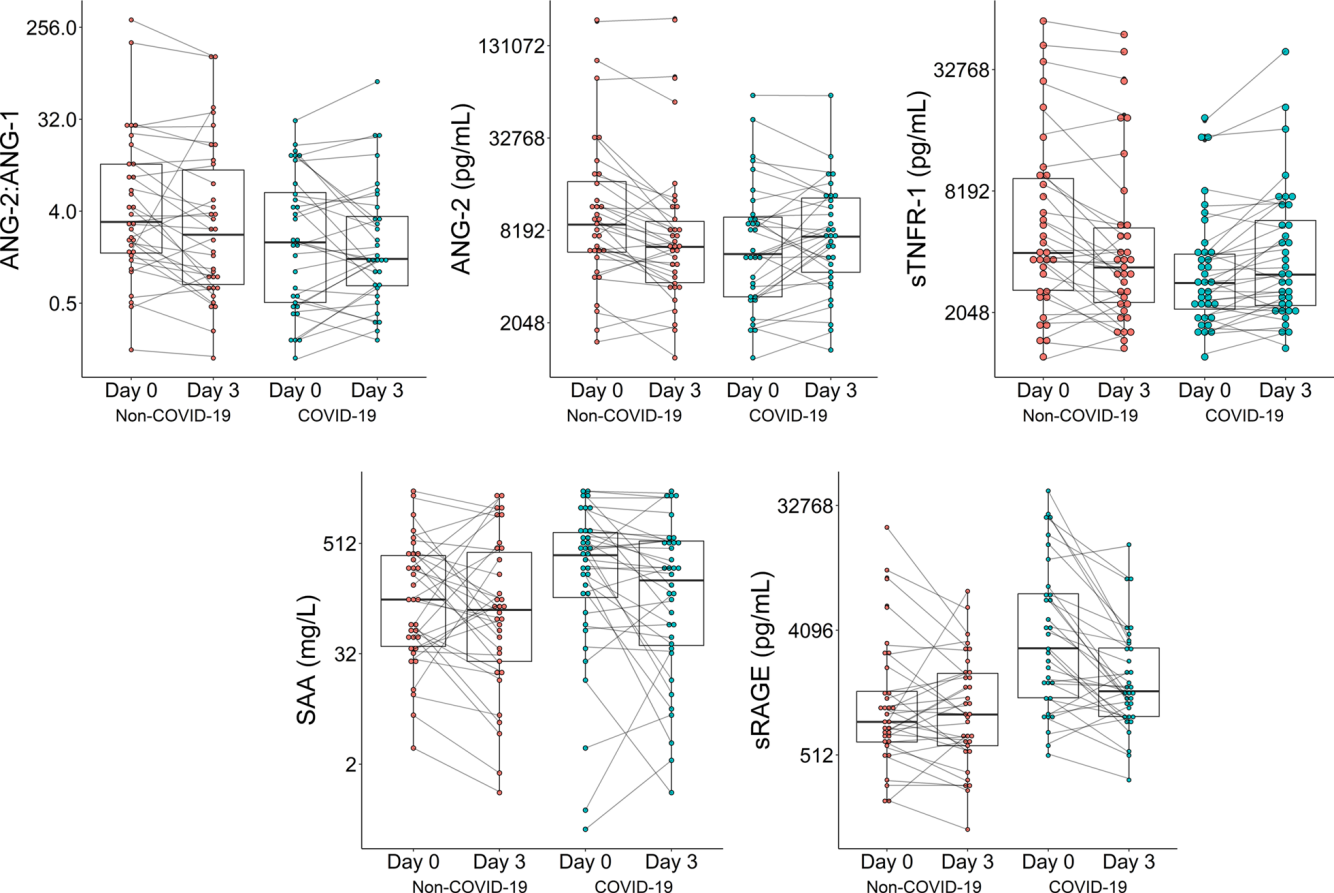
Longitudinal plasma biomarker concentrations in ICU patients with and without COVID-19 demonstrates divergent Ang-2, sTNFR-1 and sRAGE concentrations. Ang-2 and sTNFR-1 concentrations are decreasing in non-COVID-19 and increasing in COVID-19 from days 1 to 3 (*p* < 0.001 and *p* = 0.005, respectively). In contrast, sRAGE concentrations are increasing in non-COVID-19 and decreasing in COVID-19 from days 1 to 3 (*p* = 0.003). The trend of biomarker concentrations is provided from days 1 to 3 of ICU admission. P-value tests whether the ratio of the fold-change from day 1 to day 3 differs between patients with and without COVID-19 (among the subset of patients with day 1 and day 3 biomarkers). Longitudinal plasma biomarker concentrations are analyzed in the patients with days 1 and 3 biomarker measurements (non-COVID-19, *n* = 34 and COVID-19, *n* = 32)

In sensitivity analyses restricted to a primary admission diagnosis of respiratory failure or pneumonia (non-COVID = 19 *n* = 68 and COVID-19 *n* = 58), we again found that Ang-2, Ang-2:1 ratio, SAA and sRAGE were significantly different between groups with the same directions of effect (Additional file 1: Table S5). We found the prior finding with sTNFR-1 was attenuated. Next, we evaluated the longitudinal change in biomarkers from ICU admission to day 3 in the subset of patients with serial blood collected. We found that Ang-2 and sTNFR-1 concentrations increased from days 1 to 3 in COVID-19, while concentrations Ang-2 and sTNFR-1 decreased in non-COVID-19 (*p* < 0.001). We also found that sRAGE concentrations decreased in COVID-19, while concentrations increased in non-COVID-19 patients (*p* = 0.005). SAA and Ang-2:1 ratio remained stable over time. (Fig. 4 and Additional file 1: Table S6).

### Distinct molecular pathways are associated with clinical outcomes in COVID-19 and non-COVID-19 patients

We next sought to determine whether five plasma biomarkers (Ang-2, Ang-2:1 ratio, SAA, sTNFR-1 and sRAGE) that were present at different levels between COVID-19 and non-COVID-19 patients were associated with clinical outcomes. We found that none of the five biomarkers were associated with hospital mortality in COVID-19 (Table 3). In contrast, higher concentrations of Ang-2:1 ratio were associated with greater risk of hospital mortality only in non-COVID-19 patients. We also found that higher concentrations of SAA were associated with lower risk of hospital mortality only in non-COVID-19.

**Table 3:** Association of plasma biomarkers with hospital mortality among ICU patients, stratified by COVID-19 status

Plasma biomarkers	COVID-19 status	Unadjusted relative risk (95% CI)	*p* value	Model 1 relative risk (95% CI)	*p* value	Model 2 relative risk (95% CI)	*p* value
SAA	Positive	1.17 (0.97, 1.41)	0.11	1.12 (0.92, 1.36)	0.25	1.12 (0.92, 1.35)	0.27
	Negative	0.90 (0.80, 1.02)	0.09	0.89 (0.80, 0.99)	0.04	0.89 (0.80, 0.99)	0.04
Ang-2:1 ratio	Positive	1.09 (0.93, 1.29)	0.28	0.98 (0.85, 1.13)	0.76	0.98 (0.85, 1.14)	0.83
	Negative	1.28 (1.08, 1.52)	0.005	1.20 (1.01, 1.43)	0.03	1.21 (1.02, 1.44)	0.03
Ang-2	Positive	1.26 (0.99, 1.61)	0.06	1.07 (0.87, 1.32)	0.50	1.08 (0.87, 1.34)	0.47
	Negative	1.29 (0.99, 1.68)	0.055	1.20 (0.91, 1.59)	0.19	1.21 (0.92, 1.60)	0.18
sRAGE	Positive	1.23 (0.96, 1.56)	0.10	1.17 (0.97, 1.41)	0.11	1.17 (0.96, 1.41)	0.12
	Negative	1.05 (0.79, 1.40)	0.72	0.97 (0.77, 1.23)	0.80	0.97 (0.77, 1.23)	0.79
sTNFR-1	Positive	1.36 (1.07, 1.72)	0.01	1.17 (0.89, 1.52)	0.26	1.18 (0.90, 1.55)	0.24
	Negative	1.31 (1.05, 1.65)	0.02	1.20 (0.93, 1.54)	0.16	1.21 (0.94, 1.55)	0.14

*SAA* serum amyloid A, *Ang-2:1 ratio* angiopoietin-2: angiopoietin-1 ratio, *Ang-2* angiopoietin-2, *sRAGE* soluble form of receptor for advanced glycation end products, *sTNFR-1* soluble tumor necrosis factor receptor-1, *APACHE III* acute physiology and chronic health evaluation, *95% CI* 95% confidence interval. Model 1 adjustment variables: age, gender, body mass index and APACHE III scores. Model 2 adjustment variables: Model 1 and Charlson comorbidity index. Relative risk estimates are for a doubling of biomarker concentrations

We then evaluated whether plasma biomarkers were associated with two common forms of organ dysfunction in COVID-19, ARDS and severe AKI. We found that higher sRAGE concentrations were associated with ARDS only in the non-COVID-19 patients, while sRAGE was not associated with ARDS in COVID-19 (Additional file 1: Table S7). In contrast, higher SAA concentrations were only associated with ARDS in COVID-19 (aRR = 1.27 (95% CI 1.10–1.48) but not non-COVID-19 (aRR = 1.02 (95% CI 0.90–1.15). None of the plasma biomarkers were differentially associated with severe AKI based on COVID-19 status. In unadjusted analyses, we found that higher Ang-2 and sTNFR-1 concentrations were associated with a greater risk of developing severe AKI during hospitalization both in COVID-19 and non-COVID-19 populations (Additional file 1: Table S8). The association of Ang-2 with severe AKI was attenuated in both groups after adjusting for APACHE III and Charlson comorbidity index, while the association of sTNFR-1 with severe AKI remained significant in both populations.

## Discussion

The pathophysiology of severe COVID-19 has been reported to be characterized by severe inflammation and vascular injury. However, the extent to which these findings are specific to COVID-19 as opposed to critical illness in general is unclear [19]. To our knowledge, this is the first study of a cohort of ICU patients admitted as PUI in which circulating biomarkers have been compared between patients that ultimately ruled in or out for COVID-19. We found distinct differences in the relationship of biomarkers based on COVID-19 status. For example, in non-COVID-19, inflammatory and endothelial biomarkers were highly correlated and consistent with our prior work [5]. In contrast, in COVID-19 inflammatory and endothelial pathways seemed to be uncoupled. Moreover, in COVID-19, there was a lower correlation between coagulation (INR and platelets) and endothelial markers compared to non-COVID-19.

In adjusted comparisons between COVID-19 and non-COVID-19 patients, we found that circulating markers of endothelial dysfunction and inflammation (Ang-2:1 ratio, Ang-2 and sTNFR-1) were lower in COVID-19 compared to non-COVID-19 patients. In contrast, acute phase proteins (SAA), and markers of epithelial cell injury/innate immune activation (sRAGE) were significantly greater in COVID-19, independent of demographics and ICU severity of illness. While mortality rates were similar between groups, dysregulation of Ang-2:1 ratio were only associated with hospital mortality in the non-COVID-19 population. In contrast, SAA was associated with ARDS only in COVID-19 patients. Despite higher sRAGE concentrations in COVID-19 patients, higher sRAGE concentrations were associated with ARDS only in the non-COVID-19 population. These findings suggest that, unlike previously studied causes of critical illness, endothelial dysfunction may not be a distinct signature of the host response to COVID-19 infection, and, in contrast, epithelial cell injury/innate immune activation and inflammation may be a pathway for future study and targeting of therapeutics in COVID-19.

We found that rates of hospital mortality and organ dysfunction were similar in critically ill patients with and without COVID-19. These findings differ from previous studies reporting extremely high rates of AKI, thrombosis and death in patients with COVID-19 [20]. However, the study designs used in these prior reports comparing COVID-19 clinical outcomes to historical cohorts rather than within a contemporaneous cohort may result in significant confounding. Comparison of clinical outcomes between COVID-19 and a contemporaneous cohort mitigates, though does not eliminate, confounding due to differences in processes of care, differences in practice patterns due to system strains and allocation of limited resources. An additional factor differentiating our study from multiple prior studies is that while shortages of medical resources and ICU beds occurred in a number of other cities and countries, our center did not experience a limitation of resources, staff, or ICU beds and never approached crisis standards of care [21]. In addition, enrollment in the CHROME study occurred from April to September allowing for a broader representation of demographics and comorbidities among both the COVID-19 and non-COVID-19 admissions [22]. In contrast to similarities in mortality and organ dysfunction, COVID-19 patients experienced increased LOS, a very important factor that have strained ICU resources across the globe as a result of the COVID-19 pandemic.

Studies have suggested that COVID-19 is a hyperinflammatory syndrome or “cytokine storm”. The presumed elevations in IL-6 concentrations have been hypothesized as causal in severe COVID-19 and this has led to interventional trials testing therapies that block IL-6 [23, 24]. Of note, in our study, plasma concentrations of IL-6 and IL-8 were consistently elevated but similar between ICU patients with and without COVID-19 suggesting that there is heterogeneity in the inflammatory response and sub-groups of patients regardless of COVID-19 status may have elevated IL-6 concentrations. Another key inflammatory biomarker measured in our study was sTNFR-1. sTNFR-1 is the circulating form of the membrane-bound receptor which is essential for TNF signaling that results in both inflammation and endothelial dysfunction [25, 26] and we and others have shown that sTNFR-1 is highly associated with organ dysfunction and death in sepsis and other forms of ARDS [16]. Counter to what we had hypothesized, we found that sTNFR-1 was lower in COVID-19 relative to non-COVID-19, but our findings are consistent with results from Sinha et al. showing that sTNFR-1 concentrations were lower in patients with COVID-19-associated ARDS compared to a historical cohort of patients with ARDS [27].

In contrast to these prototypical inflammatory cytokines, chemokines, and cytokine receptors we found that the acute phase protein, SAA, was markedly elevated in COVID-19. Our results extend a prior report from China that demonstrated that SAA concentrations were associated with severity of respiratory failure and mortality in hospitalized patients with COVID-19 [28]. Finally, levels of sRAGE, a marker of alveolar type 1 cell-injury that is associated with risk for ARDS and related mortality, were significantly greater in COVID-19 patients [29, 30]. Our findings suggest that SAA and sRAGE may be useful indications of COVID-19 pathophysiology.

Considerable interest has focused on the potential role for vascular pathology in the development of severe COVID-19 [7]. However, in our survey of multiple markers of endothelial function/dysfunction and injury, we only observed significantly lower Ang-2 and Ang-2:1 ratio in COVID-19 relative to non-COVID-19 patients. We also found a trend toward lower sTie-2 and higher concentrations of Ang-1 in COVID-19. Previous studies have demonstrated that Ang-1 and 2 concentrations are associated with mortality in community acquired pneumonia and sepsis [6, 31]. However, the majority of studies examining Ang-1 and Ang-2 in critical illness were likely to have had had an underlying bacterial (as opposed to viral) etiology [31, 32]. Our results suggest that endothelial dysfunction is not a dominant contributor to severe COVID-19 and, more generally, that the findings of a dysregulated Ang-Tie-2 axis in bacterial sepsis may not be extrapolated to viral pneumonia. These findings also suggest that therapeutic targeting of the Ang-Tie-2 axis early in COVID-19 critical illness as has been proposed [33, 34] may not be optimal. The increasing trajectory of Ang-2 concentrations from days 1 to 3 in COVID-19 patients are intriguing and may suggest that endothelial dysfunction may be a later finding in COVID-19.

This study had a number of strengths. First, the comparison of critically ill COVID-19 patients to contemporaneous critically ill patients with symptoms clinically suggestive of COVID-19 but who tested negative for SARS-CoV-2 reduces confounding due to differences in clinical management and resource allocation due to the COVID-19 pandemic. Second, all ICU blood samples were collected with uniform handling to minimize any bias due to sample handling issues. All biomarker measurements were completed using the same assay and laboratory procedures. Third, clinical outcomes were ascertained using a detailed electronic case report form by trained research coordinators and all patients had complete hospital follow up.

Limitations of this study include the small sample size which may have compromised our ability to detect minor differences in biomarker concentrations between critically ill patients with and without COVID-19. Nonetheless, we had adequate power to exclude the large effect sizes that have been observed in prior studies of inflammatory biomarkers such as IL-6 in COVID-19 and the Ang-Tie-2 axis in sepsis and ARDS. We were able to identify several significant novel associations in spite of this limitation. Another general limitation of our study as with many COVID-19 studies in the critically ill is that the limitations to imaging and lab evaluation due to the respiratory isolation mandated for those who tested positive for SARS-CoV-2 could lead to biases for outcomes such as diagnosis of VTE or initiation of RRT in the COVID-19 patients. However, this is unlikely to have affected the biomarker measurements of the main comparison between COVID-19/non-COVID-19. Another limitation is that corticosteroids or anti-inflammatory agents may have influenced plasma biomarker concentrations between COVID-19 and non-COVID-19 groups. To account for this, we adjusted for known corticosteroid use but residual confounding may still exist.

## Conclusions

We present data from direct comparisons of critically ill patients with COVID-19 to critically ill patients with suspicion for COVID-19 who ultimately test negative for SARS-CoV-2 infection. We find that biomarkers of endothelial dysfunction were not characteristic of the host response in COVID-19, while biomarkers of epithelial cell injury and acute phase proteins were associated with COVID-19 critical illness. Our findings may help inform identification of prognostic and predictive biomarkers for COVID-19 and identify pathways for eventual therapeutic development.

## Supplementary Information

**Additional file 1.** **Table S1**: Coefficient of variation of plasma biomarkers. **Table S2**: Admission Diagnoses within Cohorts. **Table S3**: Day 1 plasma biomarkers at study enrollment between COVID-19 and non-COVID-19. **Table S4**: Plasma biomarkers restricted to patients with a primary ICU diagnosis of pneumonia or respiratory failure. **Table S5**: Trend in plasma biomarkers over Day 1 and 3 between COVID-19 and non-COVID-19. **Table S6**: Association of baseline biomarkers with ARDS. **Table S7**: Association of baseline biomarkers with severe AKI. **Figure S1**: Similar set of plasma biomarkers are different between critically ill patients with COVID-19 compared to without COVID-19 adjusting for receipt of corticosteroids.

## Data Availability

The datasets generated during and/or analyzed during the current study are not publicly available due currently ongoing research studies, but the data are available from the corresponding author on reasonable request.

## Abbreviations

AKI: Acute kidney injury
Ang 2: Angiopoietin-2
Ang-1: Angiopoietin-1
ARDS: Acute respiratory distress syndrome
bFGF: Basic fibroblast growth factor
BMI: Body mass index
CHROME: Covid-19 host response and clinical outcomes
CRP: C-reactive protein
ICU: Intensive care unit
IL-6: Interleukin 6
IL-8: Interleukin-8
IQR: Interquartile range
IRB: Institutional Review Board
PIGF: Placental growth factor
PUIs: Persons under investigation
SAA: Serum amyloid A
SARS-CoV-2: Severe acute respiratory syndrome coronavirus 2 (SARS-CoV-2)
sFas: Soluble Fas
sFlt-1: Soluble fms-like tyrosine kinase 1
sICAM-1: Intercellular adhesion molecule 1
sRAGE: Soluble receptor for advanced glycation end products
sTie2: Soluble Tie-2
sTNFR-1: Soluble tumor necrosis factor receptor-1
sTREM-1: Soluble triggering receptor expressed by myeloid cells 1
sVCAM: Vascular cell adhesion molecule
TNF-α: Tumor necrosis factor-α
UW: University of Washington
VEGF: Vascular endothelial growth factors

## References

[1] Bhatraju PK, Ghassemieh BJ, Nichols M, Kim R, Jerome KR, Nalla AK (2020). Covid-19 in critically ill patients in the Seattle region—case series. N Engl J Med.

[2] Laing AG, Lorenc A, del Molino del Barrio I, Das A, Fish M, Monin L (2020). A dynamic COVID-19 immune signature includes associations with poor prognosis. Nat Med.

[3] Richardson S, Hirsch JS, Narasimhan M, Crawford JM, McGinn T, Davidson KW (2020). Presenting characteristics, comorbidities, and outcomes among 5700 patients hospitalized with COVID-19 in the New York city area. JAMA.

[4] Reilly JP, Wang F, Jones TK, Palakshappa JA, Anderson BJ, Shashaty MGS (2018). Plasma angiopoietin-2 as a potential causal marker in sepsis-associated ARDS development: evidence from Mendelian randomization and mediation analysis. Intensive Care Med.

[5] Bhatraju PK, Zelnick LR, Herting J, Katz R, Mikacenic C, Kosamo S (2018). Identification of acute kidney injury sub-phenotypes with differing molecular signatures and response to vasopressin therapy. Am J Respir Crit Care Med.

[6] Hahn WO, Mikacenic C, Price BL, Harju-Baker S, Katz R, Himmelfarb J (2016). Host derived biomarkers of inflammation, apoptosis, and endothelial activation are associated with clinical outcomes in patients with bacteremia and sepsis regardless of microbial etiology. Virulence.

[7] Teuwen L-A, Geldhof V, Pasut A, Carmeliet P (2020). COVID-19: the vasculature unleashed. Nat Rev Immunol.

[8] Smadja DM, Guerin CL, Chocron R, Yatim N, Boussier J, Gendron N (2020). Angiopoietin-2 as a marker of endothelial activation is a good predictor factor for intensive care unit admission of COVID-19 patients. Angiogenesis.

[9] Stahl K, Gronski PA, Kiyan Y, Seeliger B, Bertram A, Pape T (2020). Injury to the endothelial glycocalyx in critically ill patients with COVID-19. Am J Respir Crit Care Med.

[10] Thwaites R, Sanchez Sevilla Uruchurtu A, Siggins M, Liew F, Russell CD, Moore S (2020). Elevated antiviral, myeloid and endothelial inflammatory markers in severe COVID-19. Infect Dis Except HIV/AIDS.

[11] Knaus WA, Wagner DP, Draper EA, Zimmerman JE, Bergner M, Bastos PG (1991). The APACHE III prognostic system. Risk prediction of hospital mortality for critically ill hospitalized adults. Chest.

[12] Beigel JH, Tomashek KM, Dodd LE, Mehta AK, Zingman BS, Kalil AC (2020). Remdesivir for the treatment of Covid-19—final report. N Engl J Med.

[13] Ranieri VM, Rubenfeld GD, Thompson BT, Ferguson ND, Caldwell E, ARDS Definition Task Force (2012). Acute respiratory distress syndrome: the Berlin Definition. JAMA.

[14] ACUTE KIDNEY INJURY|KDIGO [Internet]. http://kdigo.org/home/guidelines/acute-kidney-injury/. Cited 3 Jun 2017.

[15] Mikacenic C, Hahn WO, Price BL, Harju-Baker S, Katz R, Kain KC (2015). Biomarkers of endothelial activation are associated with poor outcome in critical illness. PLoS ONE.

[16] Mikacenic C, Price BL, Harju-Baker S, O’Mahony DS, Robinson-Cohen C, Radella F (2017). A two-biomarker model predicts mortality in the critically ill with sepsis. Am J Respir Crit Care Med.

[17] Gupta S, Hayek SS, Wang W, Chan L, Mathews KS, Melamed ML (2020). Factors associated with death in critically ill patients with coronavirus disease 2019 in the US. JAMA Intern Med.

[18] Yadaw AS, Li Y, Bose S, Iyengar R, Bunyavanich S, Pandey G (2020). Clinical features of COVID-19 mortality: development and validation of a clinical prediction model. Lancet Digit Health.

[19] Sinha P, Matthay MA, Calfee CS (2020). Is a “Cytokine Storm” relevant to COVID-19?. JAMA Intern Med.

[20] Chan L, Chaudhary K, Saha A, Chauhan K, Vaid A, Zhao S, et al. AKI in hospitalized patients with COVID-19. J Am Soc Nephrol. 2020. https://jasn.asnjournals.org/content/early/2020/09/02/ASN.2020050615. Cited 5 Nov 15 2020. 10.1681/ASN.2020050615 PMC7894657 32883700

[21] Cobb NL, Sathe NA, Duan KI, Seitz KP, Thau MR, Sung CC (2020). Comparison of clinical features and outcomes in critically ill patients hospitalized with COVID-19 versus influenza. Ann Am Thorac Soc.

[22] Horwitz LI, Jones SA, Cerfolio RJ, Francois F, Greco J, Rudy B, Petrilli CM (2020). Trends in COVID-19 risk-adjusted mortality rates. J Hosp Med.

[23] Stone JH, Frigault MJ, Serling-Boyd NJ, Fernandes AD, Harvey L, Foulkes AS (2020). Efficacy of Tocilizumab in patients hospitalized with Covid-19. N Engl J Med.

[24] Investigators TR-C, Gordon AC, Mouncey PR, Al-Beidh F, Rowan KM, Nichol AD, et al. Interleukin-6 receptor antagonists in critically ill patients with Covid-19—preliminary report. medRxiv. 2021;2021.01.07.21249390.

[25] Cunningham PN, Dyanov HM, Park P, Wang J, Newell KA, Quigg RJ (2002). Acute renal failure in endotoxemia is caused by TNF acting directly on TNF receptor-1 in kidney. J Immunol.

[26] Xu C, Chang A, Hack BK, Eadon MT, Alper SL, Cunningham PN (2014). TNF-mediated damage to glomerular endothelium is an important determinant of acute kidney injury in sepsis. Kidney Int.

[27] Sinha P, Calfee CS, Cherian S, Brealey D, Cutler S, King C, et al. Prevalence of phenotypes of acute respiratory distress syndrome in critically ill patients with COVID-19: a prospective observational study. Lancet Respir Med. 2020. https://linkinghub.elsevier.com/retrieve/pii/S2213260020303660. Cited 15 Nov 2020. 10.1016/S2213-2600(20)30366-0 PMC7718296 32861275

[28] Li H, Xiang X, Ren H, Xu L, Zhao L, Chen X (2020). Serum Amyloid A is a biomarker of severe Coronavirus Disease and poor prognosis. J Infect.

[29] Uchida T, Shirasawa M, Ware LB, Kojima K, Hata Y, Makita K (2006). Receptor for advanced glycation end-products is a marker of type I cell injury in acute lung injury. Am J Respir Crit Care Med.

[30] Jones TK, Feng R, Kerchberger VE, Reilly JP, Anderson BJ, Shashaty MGS (2020). Plasma sRAGE acts as a genetically regulated causal intermediate in sepsis-associated acute respiratory distress syndrome. Am J Respir Crit Care Med.

[31] Gutbier B, Neuhauß A-K, Reppe K, Ehrler C, Santel A, Kaufmann J (2018). Prognostic and pathogenic role of angiopoietin-1 and -2 in pneumonia. Am J Respir Crit Care Med.

[32] Han S, Lee S-J, Kim KE, Lee HS, Oh N, Park I (2016). Amelioration of sepsis by TIE2 activation-induced vascular protection. Sci Transl Med.

[33] I-SPY COVID-19 TRIAL: an adaptive platform trial for critically ill patients—full text view—ClinicalTrials.gov. https://clinicaltrials.gov/ct2/show/NCT04488081. Cited 15 Nov 2020.

[34] Bevacizumab in severe or critically severe patients with COVID-19 pneumonia-RCT—full text view—ClinicalTrials.gov. https://clinicaltrials.gov/ct2/show/NCT04305106. Cited 15 Nov 2020.

