# Supplementary material for: Comparison of host endothelial, epithelial and inflammatory response in ICU patients with and without COVID-19: a prospective observational cohort study
Source: Crit Care. 2021 Apr 19;25:148. doi: 10.1186/s13054-021-03547-z (PMC8054255; doi:10.1186/s13054-021-03547-z)
Supplement: Supplementary file 1 — Additional file 1. Table S1: Coefficient of variation of plasma biomarkers. Table S2: Admission Diagnoses within Cohorts. Table S3: Day 1 plasma biomarkers at study enrollment between COVID-19 and non-COVID-19. Table S4: Plasma biomarkers restricted to patients with a primary ICU diagnosis of pneumonia or respiratory failure. Table S5: Trend in plasma biomarkers over Day 1 and 3 between COVID-19 and non-COVID-19. Table S6: Association of baseline biomarkers with ARDS. Table S7: Association of baseline biomarkers with severe AKI. Figure S1: Similar set of plasma biomarkers are different between critically ill patients with COVID-19 compared to without COVID-19 adjusting for receipt of corticosteroids. [file 13054_2021_3547_MOESM1_ESM.docx]

**ONLINE SUPPLEMENTARY MATERIALS**

**Title:**

**Authors**: Bhatraju PK, Morrell ED, Zenick L. et al.

**Table of Contents**

**Additional Methods**

**Table S1.** Coefficient of variation of plasma biomarkers

**Table S2**. Admission Diagnoses within Cohorts

**Table S3**. Difference in clinical biomarkers between COVID-19 and non-COVID-19 patients

**Table S4.** Day 1 plasma biomarkers at study enrollment between COVID-19 and non-COVID-19

**Table S5.** Plasma biomarkers restricted to patients with a primary ICU diagnosis of pneumonia or respiratory failure

**Table S6.** Trend in plasma biomarkers over Day 1 and 3 between COVID-19 and non-COVID-19

**Table S7.** Association of baseline biomarkers with ARDS

**Table S8.** Association of baseline biomarkers with severe AKI

**Figure S1.** Similar set of plasma biomarkers are different between critically ill patients with COVID-19 compared to without COVID-19 after adjusting for receipt of corticosteroids.

**Additional Methods**

All samples were stored at -80 degrees Celsius prior to biomarker measurement. Biomarkers were measured using a multiplex assay (Meso Scale Diagnostics [MSD], Rockville, MD). The V-Plex Vascular Injury Panel included SAA, CRP, VCAM and ICAM. The V-Plex Angiogenesis Panel included FGF, PIGF, Tie-2, VEGF-A, VEGF-C, VEGF-D, sFlt-1. Other biomarkers were measured using individual plates. The MSD assay uses patterned arrays and an electrochemiluminescence detection method, which is quantified using the MSD Quickplex SQ 120 instrument. The inter and intra-assays coefficient of variation and number of patients with values below or above the limit of detection are provided in the **Table S1**. All personnel measuring the biomarkers were blinded to clinical outcomes. Biomarkers were measured for research purposes.

**Table S1.** Coefficient of variation of plasma biomarkers

| **Biomarker** | **Intraplate CV (%)** | **Interplate CV (%)** | **Below LLOD, Day 1** | **Below LLOD, Day 3** | **Above ULOD, Day 1** | **Above ULOD, Day 3** |
| --- | --- | --- | --- | --- | --- | --- |
| bFGF | 7.4 | 8.1 | 0 | 0 | 0 | 0 |
| PIGF | 4.4 | 5.5 | 0 | 0 | 0 | 0 |
| sFlt-1 | 6.7 | 7.3 | 0 | 0 | 0 | 0 |
| Tie-2 | 14.6 | 14.4 | 0 | 0 | 0 | 0 |
| VEGF-A | 4.9 | 5.7 | 0 | 0 | 0 | 0 |
| VEGF-C | 90.5 | 127.8 | 24 | 16 | 0 | 0 |
| VEGF-D | 8.6 | 11.0 | 0 | 0 | 0 | 0 |
| Eotaxin | 23.7 | 28.4 | 0 | 0 | 0 | 0 |
| Eotaxin-3 | 14 | 14.7 | 0 | 0 | 0 | 0 |
| IL-6 | 11.4 | 12.3 | 0 | 0 | 3 | 0 |
| IL-8 | 11.1 | 10.8 | 0 | 0 | 0 | 0 |
| sFas | 12.8 | 12.4 | 0 | 0 | 0 | 0 |
| sTREM-1 | 16.1 | 14.8 | 0 | 0 | 0 | 0 |
| TNF-R1 | 14.5 | 13.9 | 0 | 0 | 8 | 6 |
| CRP | 8.6 | 10.3 | 0 | 0 | 0 | 0 |
| SAA | 17.2 | 19.8 | 0 | 0 | 0 | 0 |
| sICAM-1 | 7.8 | 7.9 | 0 | 0 | 0 | 0 |
| sVCAM-1 | 6.6 | 6.7 | 0 | 0 | 0 | 0 |
| VEGF | 8.8 | 9.9 | 0 | 0 | 0 | 0 |
| sRAGE | 8.0 | 7.8 | 0 | 0 | 0 | 0 |
| Ang-1 | 4.1 | 8.0 | 0 | 0 | 0 | 0 |
| Ang-2 | 4.5 | 8.5 | 0 | 0 | 3 | 1 |

Coefficient of variation (CV) of individual biomarkers and number of subjects above or below the limit of detection for each biomarker

LLOD – lower limit of detection

ULOD – upper limit of detection

**Table S2**. Admission Diagnoses within Cohorts

|  | **ICU COVID-19**  **Negative**  **(N = 93)** | **ICU COVID-19**  **Positive**  **(N = 78)** |
| --- | --- | --- |
| **Admission Diagnosis** |  |  |
| Pneumonia or Respiratory distress/failure | 70 (75) | 58 (74) |
| Composite of Sepsis/Septic  Shock/NSTI/Bacteremia/Cellulitis/Urinary Tract  Infection/Abscess | 22 (24) | 11 (14) |
| Sepsis | 8 (9) | 2 (3) |
| Septic Shock | 11 (12) | 4 (5) |
| Gastrointestinal Bleeding | 3 (3) | 2 (3) |
| Alcohol abuse or withdrawal or drug overdose | 3 (3) | 2 (3) |
| Asthma exacerbation or chronic obstructive  pulmonary disease exacerbation | 5 (5) | 2 (3) |
| Hypertension | 3 (3) | 4 (5) |
| Trauma | 0 (0) | 8 (10) |
| Congestive Heart Failure | 5 (5) | 1 (1) |
| Renal Failure | 1 (1) | 1 (1) |
| Stroke/CVA/intracranial hemorrhage | 0 (0) | 3 (4) |
| Myocardial infarction or chest pain or  cardiac/respiratory arrest, arrythmia | 7 (8) | 4 (5) |
| None | 8 (9) | 4 (5) |

Diagnoses are not mutually exclusive. Patients can have more than one diagnosis on ICU admission.

**Table S3**. Difference in clinical biomarkers between COVID-19 and non-COVID-19 patients

| **Characteristics** | **Missing in ICU COVID-19 Negative** | **Missing in ICU COVID-19 Positive** | **ICU COVID-19 Negative**  **(N = 93), median (IQR)** | **ICU COVID-19 Positive**  **(N = 78), median (IQR)** | **p-value** |
| --- | --- | --- | --- | --- | --- |
| 1. Lactic acid Dehydrogenase (LD), U/L | 75 | 29 | 410.7 (255.2) | 508.4 (431.2) | 0.26 |
| 1. Ferritin (FER), ng/mL | 86 | 28 | 2538.1 (4404.0) | 1521.8 (3620.7) | 0.58 |
| 1. D-dimer, mcg/mL | 75 | 37 | 6.3 (7.5) | 6.6 (11.6) | 0.92 |
| 1. White blood cell count (WBC), per mm^3^ | 1 | 1 | 18.7 (22.5) | 11.6 (6.2) | 0.004 |
| 1. Hematocrit (HCT), % | 1 | 1 | 30.7 (7.4) | 32.1 (6.8) | 0.21 |
| 1. Platelets (PLT), 10^9^/L | 1 | 1 | 160.0 (93.7) | 209.2 (113.0) | 0.003 |
| 1. Serum Bicarbonate (HCO_3_), mEq/L | 0 | 0 | 22.6 (6.3) | 23.3 (5.4) | 0.45 |
| 1. Serum Creatinine (Cr), mg/dL | 0 | 0 | 2.0 (2.2) | 1.7 (2.1) | 0.36 |
| 1. Aspartate aminotransferase (AST), U/liter | 10 | 14 | 126.0 (376.3) | 133.1 (400.3) | 0.91 |
| 1. Alanine aminotransferase (ALT), U/liter | 10 | 13 | 72.1 (153.6) | 78.1 (191.3) | 0.84 |
| 1. Prothrombin time (PT), seconds | 19 | 7 | 17.4 (4.6) | 16.4 (6.1) | 0.25 |
| 1. International normalized ratio (INR) | 19 | 7 | 1.5 (0.5) | 1.4 (0.7) | 0.33 |
| 1. Troponin, ng/mL | 35 | 18 | 1.0 (2.2) | 0.4 (1.7) | 0.13 |

P-value is based on a two sample t-test assuming unequal variances

**Table S4.** Day 1 plasma biomarkers at study enrollment between COVID-19 and non-COVID-19

| **Plasma biomarkers, pg/mL** | **ICU COVID-19 Negative**  **(N = 93)** | **ICU COVID-19 Positive**  **(N = 78)** | **Unadjusted Fold-change between COVID-19 vs non COVID-19**  **(95% CI)** | ***p-value*** | **Adjusted Fold-change between COVID-19 vs non COVID-19**  **(95% CI)** | ***p-value*** |
| --- | --- | --- | --- | --- | --- | --- |
| 1. SAA, mg/L | 75 (28-380) | 335 (70-677) | 2.92 (1.62, 5.26) | 0.0004 | **2.94 (1.67, 5.18)** | **0.0002** |
| 1. sRAGE | 1526 (762-2754) | 2684 (1191-6428) | 1.82 (1.31, 2.53) | 0.0004 | **1.89 (1.35, 2.65)** | **0.0002** |
| 1. CRP, mg/L | 96 (39-260) | 150 (84-305) | 1.77 (1.15, 2.74) | 0.01 | 1.88 (1.20, 2.95) | 0.006 |
| 1. Ang-1 | 1927 (810-4630) | 2971 (1561-7115) | 1.74 (1.24, 2.44) | 0.001 | 1.58 (1.13, 2.22) | 0.008 |
| 1. BFGF | 7 (4-18) | 12 (8-20) | 1.60 (1.19, 2.13) | 0.002 | 1.48 (1.12, 1.96) | 0.006 |
| 1. Eotaxin-3 | 23 (14-33) | 28 (19-41) | 1.40 (1.07, 1.84) | 0.01 | 1.35 (1.04, 1.76) | 0.02 |
| 1. sVCAM-1, ng/mL | 1200 (770-1500) | 1100 (883-1400) | 1.11 (0.81, 1.54) | 0.51 | 1.15 (0.84, 1.58) | 0.39 |
| 1. sICAM-1, ng/mL | 880 (590-1300) | 880 (642-1175) | 1.07 (0.91, 1.25) | 0.43 | 1.13 (0.96, 1.32) | 0.14 |
| 1. IL-6 | 24 (10-163) | 37 (15-92) | 0.93 (0.51, 1.72) | 0.83 | 1.16 (0.64, 2.12) | 0.62 |
| 1. Eotaxin | 211 (141-291) | 240 (147-357) | 1.11 (0.91, 1.37) | 0.31 | 1.10 (0.90, 1.34) | 0.37 |
| 1. sFLT-1 | 264 (169-647) | 292 (164-507) | 0.89 (0.65, 1.21) | 0.45 | 1.01 (0.77, 1.33) | 0.94 |
| 1. sFAS | 13522 (10846-19901) | 13927 (9958-18842) | 1.01 (0.83, 1.23) | 0.92 | 1.02 (0.85, 1.23) | 0.85 |
| 1. PIGF | 20 (13-28) | 18 (14-22) | 1.01 (0.83, 1.23) | 0.91 | 0.99 (0.81, 1.21) | 0.94 |
| 1. VEGF-C | 80 (5-150) | 63 (8-152) | 0.98 (0.55, 1.74) | 0.95 | 0.92 (0.52, 1.63) | 0.76 |
| 1. VEGF-D | 1454 (964-1799) | 1259 (907-1797) | 0.94 (0.81, 1.09) | 0.39 | 0.91 (0.79, 1.05) | 0.19 |
| 1. Tie-2 | 3967 (3305-4832) | 3607 (3079-4239) | 0.88 (0.81, 0.96) | 0.006 | 0.88 (0.81, 0.97) | 0.008 |
| 1. VEGF-A | 148 (80-290) | 133 (90-241) | 0.96 (0.67, 1.37) | 0.82 | 0.88 (0.61, 1.27) | 0.49 |
| 1. TNF-alpha | 3.41 (1.68-5.31) | 3.19 (2.10-4.40) | 0.83 (0.62, 1.11) | 0.20 | 0.87 (0.64, 1.17) | 0.36 |
| 1. sTREM-1 | 436 (258-846) | 351 (211-596) | 0.75 (0.57, 0.99) | 0.04 | 0.78 (0.62, 0.98) | 0.03 |
| 1. TNF-RI | 4558 (2647-12815) | 3089 (1985-5480) | 0.61 (0.45, 0.81) | 0.0007 | **0.63 (0.48, 0.83)** | **0.001** |
| 1. IL-8 | 511 (3-1035) | 392 (3-802) | 0.64 (0.29, 1.42) | 0.27 | 0.61 (0.27, 1.37) | 0.23 |
| 1. Ang-2 | 8844 (4988-19910) | 5268 (2833-9338) | 0.55 (0.41, 0.74) | < 0.0001 | **0.61 (0.45, 0.82)** | **0.001** |
| 1. Ang-2:1 ratio | 4.7 (1.6-16.2) | 1.6 (0.5-4.8) | 0.32 (0.20, 0.51) | < 0.0001 | **0.38 (0.24, 0.62)** | **< 0.0001** |

Abbreviations: angiopoietin-1 (Ang-1), angiopoietin-2 (Ang-2), Ang-2:1 ratio, angiopoietin-2: angiopoietin-1 ratio, basic fibroblast growth factor (bFGF), placental growth factor (PIGF), soluble fms-like tyrosine kinase 1 (sFlt-1), soluble Tie-2, vascular endothelial growth factors A, C, D, eotaxin-1, eotaxin-3, intercellular adhesion molecule 1 (sICAM-1), vascular cell adhesion molecule (sVCAM), interleukin-6 (IL-6), interleukin-8 (IL-8), soluble tumor necrosis factor receptor-1 (sTNFR-1), tumor necrosis factor-α (TNF-α), c-reactive protein (CRP), serum amyloid A (SAA), soluble receptor for advanced glycation end products (sRAGE), soluble Fas (sFAS) and soluble triggering receptor expressed by myeloid cells 1 (sTREM-1).

Adjusted for age, gender, body mass index and APACHE III scores. Fold change greater than 1 means higher plasma biomarker concentrations in the COVID-19 compared to the non-COVID-19 population. Bonferroni corrected *p-value* for significance of 0.002

**Table S5.** Plasma biomarkers restricted to patients with a primary ICU diagnosis of pneumonia or respiratory failure

| **Plasma biomarkers, pg/mL** | **ICU COVID-19 Negative**  **(N = 70)**  **Median (IQR)** | **ICU COVID-19 Positive**  **(N = 58)**  **Median (IQR)** | **Fold-change between COVID-19 vs non COVID-19 (95% CI)** | **p-value** |
| --- | --- | --- | --- | --- |
| Ang-2 | 8296 (4669-16162) | 5411 (3021-9243) | 0.71 (0.52, 0.98) | 0.04 |
| Ang-2:1 ratio | 4.0 (1.7-12.9) | 1.7 (0.5-4.4) | 0.41 (0.24, 0.69) | 0.0008 |
| sTNF-RI | 4401 (2697-12302) | 3381 (2068-5480) | 0.67 (0.50, 0.92) | 0.01 |
| SAA, mg/L | 77 (35-375) | 395 (167-792) | 3.41 (1.84, 6.31) | < 0.0001 |
| sRAGE | 1479 (724-2651) | 3140 (1600-7273) | 2.21 (1.47, 3.33) | 0.0001 |

Abbreviations: angiopoietin-2 (Ang-2), Ang-2:1 ratio, angiopoietin-2:angiopoietin-1 ratio; soluble tumor necrosis factor receptor-1 (sTNFR-1), serum amyloid A (SAA) and soluble receptor for advanced glycation end products (sRAGE).

Adjusted for age, gender, body mass index and APACHE III scores. Fold change greater than 1 means higher plasma biomarker concentrations in the COVID-19 compared to the non-COVID-19 population.

**Table S6.** Trend in plasma biomarkers over Day 1 and 3 between ICU patients with COVID-19 and without COVID-19

| **Plasma biomarkers, pg/dL** | **Day 1 plasma biomarkers in ICU COVID-19 Negative**  **(N = 33)**  **Median (IQR)** | **Day 3 plasma biomarkers in ICU COVID-19 Negative**  **(N = 33)**  **Median (IQR)** | **Day 1 plasma biomarkers in ICU COVID-19 Positive**  **(N = 32)**  **Median (IQR)** | **Day 3 plasma biomarkers in ICU COVID-19 Positive**  **(N = 32)**  **Median (IQR)** | **p-value** |
| --- | --- | --- | --- | --- | --- |
| 1. Ang-2 | 8934 (5904-17020) | 6510 (4044-9702) | 5743 (3021-9988) | 7461 (4369-13319) | 0.0006 |
| 2. Ang-2:1 ratio | 3.1 (1.6-11.6) | 2.5 (0.9-11.2) | 2.0 (0.5-6.2) | 1.4 (0.7-3.6) | 0.27 |
| 3. sTNFRI | 3933 (2632-8827) | 3292 (2281-5537) | 2908 (2194-4170) | 3303 (2315-7017) | < 0.0001 |
| 4. SAA, mg/L | 120 (36-340) | 93 (25-430) | 405 (134-695) | 275 (67-550) | 0.98 |
| 5. sRAGE | 889 (635-1479) | 1003 (581-1905) | 2954 (1318-7303) | 1523 (969-3116) | 0.005 |

Abbreviations: angiopoietin-2 (Ang-2), Ang-2:1 ratio, angiopoietin-2:angiopoietin-1 ratio; soluble tumor necrosis factor receptor-1 (sTNFR-1), serum amyloid A (SAA) and soluble receptor for advanced glycation end products (sRAGE). P-value tests whether the ratio of the fold-change from Day 1 to Day 3 differs between patients with and without COVID-19 (among the subset of patients with Day 1 and Day 3 biomarkers). Analysis is restricted to patients with days 1 and 3 blood samples.

**Table S7.** Association of baseline biomarkers with ARDS

| **Plasma Biomarkers** | **COVID-19 Status** | **Unadjusted Relative Risk (95% CI)** | ***p-value*** | **Model 1 Relative Risk (95% CI)** | ***p-value*** | **Model 2 Relative Risk (95% CI)** | ***p-value*** |
| --- | --- | --- | --- | --- | --- | --- | --- |
| **Ang-2** | **Positive** | 1.14 (0.92, 1.40) | 0.23 | 0.91 (0.72, 1.14) | 0.41 | 0.95 (0.76, 1.19) | 0.67 |
|  | **Negative** | 1.15 (0.94, 1.42) | 0.18 | 1.05 (0.88, 1.24) | 0.59 | 1.07 (0.88, 1.31) | 0.49 |
|  |  |  |  |  |  |  |  |
| **Ang-2:1 ratio** | **Positive** | 1.08 (0.94, 1.23) | 0.27 | 0.96 (0.84, 1.10) | 0.57 | 1.01 (0.89, 1.15) | 0.84 |
|  | **Negative** | 1.08 (0.94, 1.26) | 0.29 | 1.05 (0.92, 1.19) | 0.49 | 1.08 (0.94, 1.24) | 0.30 |
|  |  |  |  |  |  |  |  |
| **sRAGE** | **Positive** | 1.00 (0.83, 1.21) | 0.99 | 0.99 (0.82, 1.20) | 0.93 | 0.97 (0.82, 1.14) | 0.70 |
|  | **Negative** | 1.45 (1.17, 1.78) | 0.0005 | 1.34 (1.11, 1.62) | 0.002 | 1.34 (1.08, 1.66) | 0.008 |
|  |  |  |  |  |  |  |  |
| **sTNFR-1** | **Positive** | 1.01 (0.81, 1.27) | 0.92 | 0.74 (0.57, 0.95) | 0.02 | 0.80 (0.63, 1.02) | 0.07 |
|  | **Negative** | 1.16 (0.94, 1.43) | 0.17 | 1.02 (0.86, 1.21) | 0.80 | 1.07 (0.89, 1.30) | 0.47 |
|  |  |  |  |  |  |  |  |
| **SAA** | **Positive** | 1.31 (1.13, 1.51) | 0.0004 | 1.33 (1.12, 1.56) | 0.0008 | 1.27 (1.10, 1.48) | 0.001 |
|  | **Negative** | 1.02 (0.90, 1.16) | 0.74 | 1.04 (0.93, 1.16) | 0.51 | 1.02 (0.90, 1.15) | 0.74 |

Abbreviations: SAA, serum amyloid A; Ang-2:1 ratio, angiopoietin-2:angiopoietin-1 ratio; Ang-2, angiopoietin-2; sRAGE, soluble form of receptor for advanced glycation end products; sTNFR-1, soluble tumor necrosis factor receptor-1, APACHE III, acute physiology and chronic health evaluation; 95% CI, 95% confidence interval.

Model 1 adjustment variables: age, gender, body mass index and APACHE III scores. Model 2 adjustment variables: Model 1 and Charlson comorbidity index. Relative risk estimates are for a doubling of biomarker concentrations.

**Table S9.** Association of baseline biomarkers with severe AKI

| **Plasma Biomarkers** | **COVID-19 Status** | **Unadjusted Relative Risk (95% CI)** | ***p-value*** | **Model 1 Relative Risk (95% CI)** | ***p-value*** | **Model 2 Relative Risk (95% CI)** | ***p-value*** |
| --- | --- | --- | --- | --- | --- | --- | --- |
| **Ang-2** | **Positive** | 1.47 (1.03, 2.10) | 0.03 | 1.37 (0.88, 2.11) | 0.16 | 1.45 (0.89, 2.38) | 0.14 |
|  | **Negative** | 1.40 (1.08, 1.82) | 0.01 | 1.29 (0.99, 1.68) | 0.06 | 1.33 (0.99, 1.79) | 0.055 |
|  |  |  |  |  |  |  |  |
| **Ang-2:1 ratio** | **Positive** | 1.14 (0.87, 1.50) | 0.34 | 1.10 (0.81, 1.49) | 0.55 | 1.13 (0.81, 1.58) | 0.45 |
|  | **Negative** | 1.19 (0.90, 1.59) | 0.23 | 1.18 (0.91, 1.52) | 0.21 | 1.22 (0.92, 1.61) | 0.16 |
|  |  |  |  |  |  |  |  |
| **sRAGE** | **Positive** | 1.21 (0.86, 1.70) | 0.26 | 1.20 (0.84, 1.71) | 0.31 | 1.18 (0.84, 1.68) | 0.34 |
|  | **Negative** | 0.83 (0.56, 1.23) | 0.37 | 0.80 (0.54, 1.19) | 0.27 | 0.79 (0.53, 1.18) | 0.26 |
|  |  |  |  |  |  |  |  |
| **sTNFR-1** | **Positive** | 2.07 (1.43, 2.99) | 0.0001 | 1.98 (1.17, 3.33) | 0.01 | 2.14 (1.27, 3.59) | 0.004 |
|  | **Negative** | 1.69 (1.18, 2.44) | 0.005 | 1.71 (1.09, 2.68) | 0.02 | 1.77 (1.11, 2.81) | 0.02 |
|  |  |  |  |  |  |  |  |
| **SAA** | **Positive** | 1.10 (0.89, 1.36) | 0.39 | 1.08 (0.84, 1.38) | 0.57 | 1.06 (0.83, 1.36) | 0.64 |
|  | **Negative** | 1.01 (0.79, 1.28) | 0.96 | 1.02 (0.80, 1.29) | 0.89 | 1.01 (0.79, 1.29) | 0.92 |

Abbreviations: SAA, serum amyloid A; ANG-2:1 ratio, angiopoietin-2:angiopoietin-1 ratio; ANG-2, angiopoietin-2; sRAGE, soluble form of receptor for advanced glycation end products; sTNFR-1, soluble tumor necrosis factor receptor-1, APACHE III, acute physiology and chronic health evaluation; 95% CI, 95% confidence interval.

Model 1 adjustment variables: age, gender, body mass index and APACHE III scores. Model 2 adjustment variables: Model 1 and Charlson comorbidity index. Relative risk estimates are for a doubling of biomarker concentrations.

**Figure S1.** Similar set of plasma biomarkers are different between critically ill patients with COVID-19 compared to without COVID-19 after adjusting for receipt of corticosteroids.

Volcano plot on the left displays difference in plasma biomarkers between COVID-19 and non-COVID-19. Dashed line indicates Bonferroni-corrected threshold; dotted line indicates nominal 5% significance threshold. Blue dots represent biomarker concentrations that are lower in COVID-19 and gray represent concentrations higher in COVID-19. Estimates are adjusted for age, sex, BMI, and APACHE III score. Right display additionally adjusted for receipt of 6 mg of dexamethasone or equivalent prior to plasma collection. **
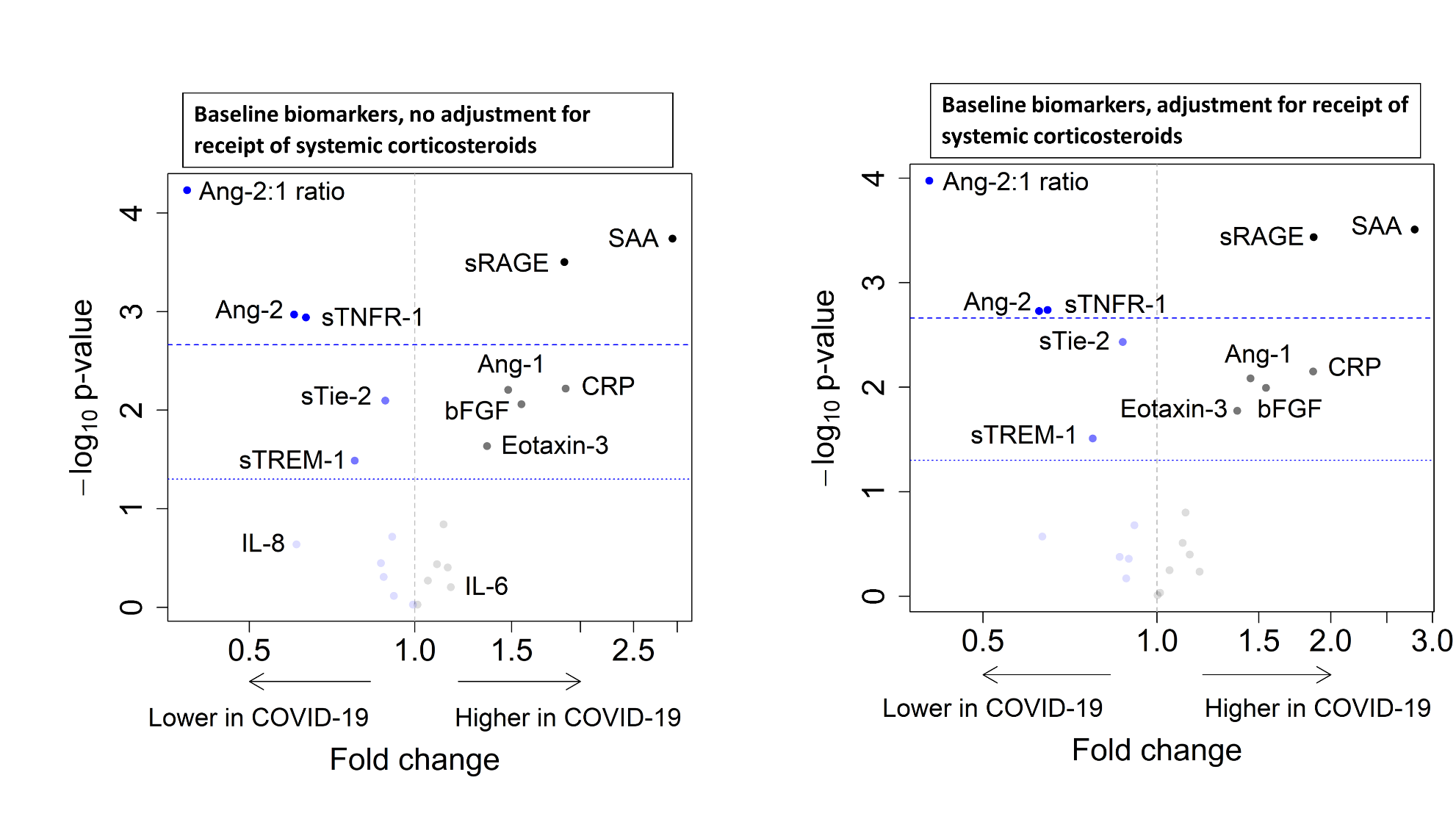
**
